# Enzootic *Angiostrongylus* *cantonensis* in Rats and Snails after an Outbreak of Human Eosinophilic Meningitis, Jamaica

**DOI:** 10.3201/eid0803.010316

**Published:** 2002-03

**Authors:** John F. Lindo, Cecilia Waugh, John Hall, Colette Cunningham-Myrie, Deanna Ashley, Mark L. Eberhard, James J. Sullivan, Henry S. Bishop, David G. Robinson, Timothy Holtz, Ralph D. Robinson

**Affiliations:** * University of the West Indies, Jamaica; †Ministry of Health, Kingston, Jamaica; ‡ Centers for Disease Control and Prevention, Atlanta, GA, USA; § Animal and Plant Health Inspection Service, US Department of Agriculture, Philadelphia, PA, USA

**Keywords:** *Angiostrongylus* *cantonensis*, rats, snails, Jamaica

## Abstract

After an outbreak in 2000 of eosinophilic meningitis in tourists to Jamaica, we looked for *Angiostrongylus cantonensis* in rats and snails on the island. Overall, 22% (24/109) of rats harbored adult worms, and 8% (4/48) of snails harbored *A. cantonensis* larvae. This report is the first of enzootic *A. cantonensis* infection in Jamaica, providing evidence that this parasite is likely to cause human cases of eosinophilic meningitis.

*Angiostrongylus cantonensis* is the most common infectious cause of eosinophilic meningitis worldwide (*1*). Although human infections with *A. cantonensis* are traditionally associated with Southeast Asia and the Pacific Basin, sporadic cases have been reported in several countries outside this region (*1*,*2*). In the Caribbean, eosinophilic meningitis has not been commonly reported, although *A. cantonensis* has been found in rats from Cuba, Puerto Rico, and the Dominican Republic (*3*–*5*).

A case of eosinophilic meningitis was described in 1994 in an adult Jamaican who had never traveled outside the country (*6*,*7*). In the absence of confirmatory histology or serology, the question of the endemicity of *A. cantonensis* in Jamaica at that time was raised (*6*,*7*). In May 2000, 12 persons in a group of 23 U.S. tourists who visited Jamaica for a week met the clinical definition for eosinophilic meningitis within 6-30 days (median 11) of returning home. Nine persons required hospitalization; there were no deaths. There was serologic evidence of exposure to *A. cantonensis* in eight persons who had eaten salad at the same restaurant, a common exposure that might account for all cases.

Since *A. cantonensis* has not been documented in Jamaica and many restaurants in Jamaica’s tourist areas serve imported vegetables, the source of contamination of the vegetables was not necessarily on the island. We investigated whether *A. cantonensis* occurs naturally in the wild rat and snail populations of Jamaica.

The Ministry of Health collected 109 rats through the rat control program run by the Public Health Department. Rats were collected in eight sites across the island (Table) and sent to the Parasitology Research Laboratories at the University of the West Indies, where the cardiopulmonary system was dissected to determine infection status. In addition, staff from University of the West Indies and the Centers for Disease Control and Prevention (CDC) collected snails from four sites (Table) and examined them for infection.

**Table:** Recovery of *Angiostrongylus* *cantonensis* from rats and snails, Jamaica, 2000

Location	Host	No. infected/no. examined (%)
Rats		
Freeport	*Rattus norvegicus* *R.rattus*	6/10 (60) 0/0
Mandeville	*R. norvegicus* *R. rattus*	10/17 (59) 0/0
Black River	*R. norvegicus* *R.rattus*	2/11 (18) 1/4 (25)
Kingston	*R. norvegicus* *R.rattus*	1/12 (8) 1/11 (9)
Lucea	*R. norvegicus* *R.rattus*	1/15 (7) 0/0
Montego Bay	*R. norvegicus* *R.rattus*	0/0 1/12 (8)
Port Antonio	*R. norvegicus* *R.rattus*	0/8 0/3
Lime Hall	*R. norvegicus* *R.rattus*	0/1 1/1 (100)
Snails		
Mandeville	*Thelidomus asper*	4/10 (40)
Brown’s Town	*Orthalicus jamaicensis Dentellaria sloaneana*	0/27 0/2
Yallahs	*Orthalicus jamaicensis*	0/6
Scott’s Pass	*Orthalicus jamaicensis*	0/3

Adult worms were recovered from the cardiopulmonary systems of 24 rats (20/78 *Rattus norvegicus*; 4/31 *R. rattus*) (Table). These worms had features characteristic of *Angiostrongylus*, including size (males measured 14-15 mm in length; females 24-26 mm in length), body shape, and prominent dark intestine (Figure 1A). The long copulatory spicules in the male worms, which measured approximately 1.2 mm (Figure 1B), are diagnostic for *A. cantonensis*, as the spicules of other species in the genus are generally <0.5 mm long (*8*).

**Figure 1:**
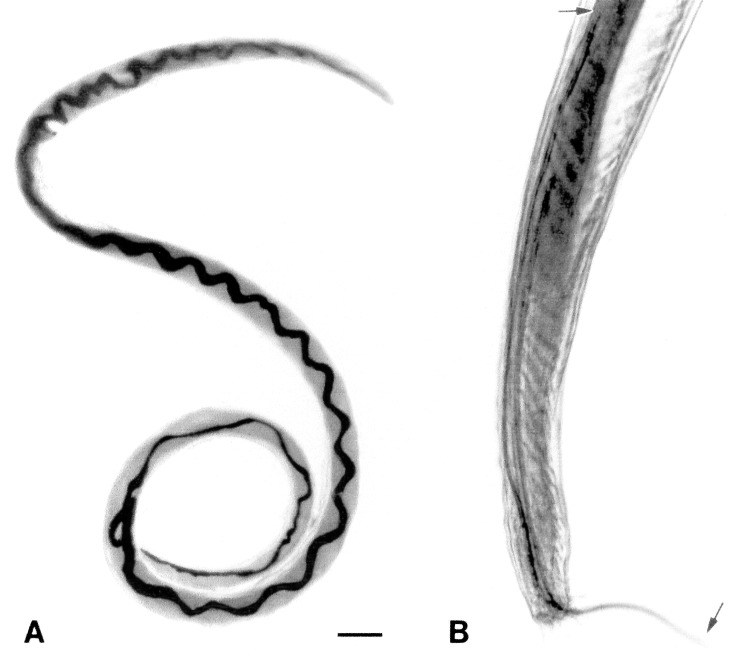
Adult *Angiostrongylus* *cantonensis* recovered from rat lungs. A. Adult female worm with characteristic barber-pole appearance (anterior end of worm is to the top). Scale bar = 1 mm. B. Tail of adult male, showing copulatory bursa and long spicules (arrows). Scale bar = 85 μm.

Overall, 22% of the rats were infected with *A. cantonensis*. Infection rates did not differ significantly between *R. rattus* and *R. norvegicus* (chi square 2.10; p=0.148). The mean number of worms recovered per infected rat was 17±3.5 (range 3-27).

Land snails (Figure 2) were collected by hand from small farms and residential gardens and sent to the Division of Parasitic Diseases laboratory, CDC, Atlanta. A portion of the muscular head-foot region was excised from each surviving snail, cut into smaller fragments, and placed in separate dishes containing digestion fluid (0.01% pepsin in 0.7% v/v aqueous HCl [*9*]). Dishes were examined for nematode larvae at 4-5 hours and 24 hours after digestion.

**Figure 2:**
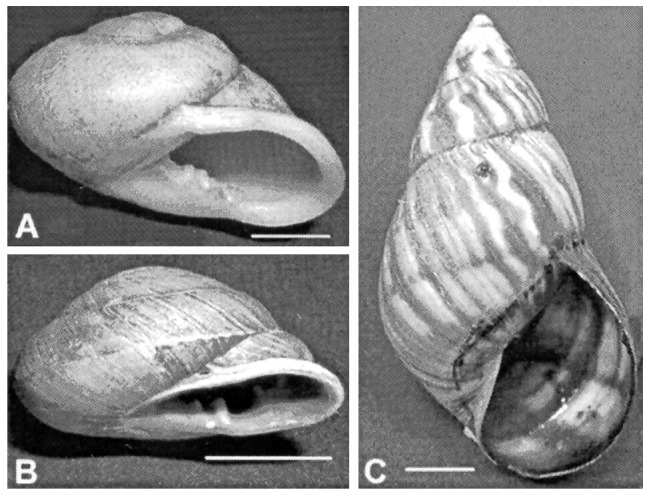
Three species of land snails collected in Jamaica and examined for *Angiostrongylus* larvae. A. *Thelidomus asper*. B. *Orthalicus jamaicensis*. C. *Dentellaria* *sloaneana*. Scale bar = 1 cm.

Four of 10 *Thelidomus asper* collected in Mandeville were found positive for *A. cantonensis* larvae, but neither *Orthalicus jamaicensis* (n=36) nor *Dentellaria sloaneana* (n=2) were infected (Table). Living larvae digested from *Thelidomus* were easily recognized and recovered because they retained motility in the digestion fluid. Larvae were examined microscopically, and the morphologic features compared with those in published reports (*10*) and reference *A*. *cantonensis* larvae to confirm identification. Two species of lungworm (metastrongyles) larvae were recovered. Most larvae were *Angiostrongylus cantonensis* (375 to 420 [mean 402] µm in length after fixation in hot alcohol), but a small number of *Aelurostrongylus abstrusus* (400 to 440 [mean 427] µm in length after fixation in hot alcohol), a lungworm of cats, were also observed. Typical of lungworm larvae, the two species were similar in size and the presence of characteristic sclerotized rhabdions at the anterior end of the larvae. The larvae were easily distinguished, however, by the shape of the tip of the tail; *A. cantonensis* had a constriction near the end of the tail and ended in a fine point, while *A. abstrusus* terminated in a knob (*10*,*11*).

This is the first report of enzootic *A. cantonensis* infection in Jamaican rats and snails; our data show that the range of the parasite extends to another Caribbean country outside Cuba, the Dominican Republic, and Puerto Rico (*3*–*5*). The occurrence of the parasite at high rates in rats and in specific groups of snails, earlier findings of eosinophilic meningitis in a resident, and the recent outbreak of *A. cantonensis*-associated eosinophilic meningitis in visitors to the island suggest that autochthonous transmission to humans is probable in Jamaica. These studies are being extended to determine the full distribution of the parasite and the species of snails involved in its transmission. Furthermore, serologic tests need to be developed to confirm infections in persons in the Caribbean.

Public health officials, clinical parasitologists, and travel medicine practitioners should consider *A. cantonensis* as a causative agent of eosinophilic meningitis in Jamaican residents and travelers to the island.
